# Severe Neurological Complications With Influenza in Vietnamese Children

**DOI:** 10.1111/irv.70035

**Published:** 2024-11-04

**Authors:** Sy Duc Nguyen, Thi Huyen Trang Ngo, Thi Viet Ha Nguyen, Thien Hai Do

**Affiliations:** ^1^ Department of Pediatrics Hanoi Medical University Hanoi Vietnam; ^2^ Vietnam National Children's Hospital Hanoi Vietnam; ^3^ Pediatrics Ward Hanoi Medical University Hospital Hanoi Vietnam

**Keywords:** children, encephalitis, influenza, influenza‐associated neurological complications (IANCs)

## Abstract

**Background:**

Influenza is a common contagious respiratory virus that primarily causes respiratory tract infections. Neurological complications associated with influenza have also been reported, mainly in pediatric populations, and may be fatal.

**Methods:**

A descriptive study evaluated pediatric patients who were diagnosed with severe influenza‐associated neurological complications at the Tropical Pediatrics Center—Vietnam National Children's Hospital from October 2022 to February 2024.

**Results:**

In this study involving 20 patients, 80% of children were under 5 years old; 70% of patients had a history of good health. All patients had not received an influenza vaccination within 12 months. The median time from onset to neurological symptoms was 1 day. The most common neurological complication was encephalitis (16/20 patients) with symptoms included altered consciousness and seizures. Most patients had elevated levels of ALT (60%), AST (90%), LDH (94%), and ferritin (69%) in serum. The imaging of brain damage on MRI and CT scans varied in patterns and locations. There was no difference in the timing of methylprednisolone treatment within and after 48 h. The mortality rate was 20%, with 45% of patients experiencing severe sequelae.

**Conclusions:**

IANCs are severe with damage to both white matter and central gray matter and can occur in healthy children, emphasizing the importance of vaccination to reduce the risk.

## 1 Introduction

Influenza is a common contagious respiratory virus that primarily causes respiratory tract infections. It can occur throughout the year in all countries around the world, but predominantly during the winter. Influenza virus can infect susceptible population of all ages, yet the infections rates are highest among children, affecting an average of 20%–30% annually [1]. Symptoms associated with influenza are very diverse, ranging from mild infections, such as cough and runny nose, to severe conditions like pneumonia, cardiac, neurological, and hematologic complications. Certain people may be at increased risk for developing flu‐related complications, including children younger than 5 years old, those with medical conditions such as chronic respiratory, cardiovascular, renal, hepatic, neurological, hematologic disorders, or metabolic disorders; immunocompromised children due to medication or illness; and children undergoing prolonged Aspirin therapy who are at risk of developing Reye's syndrome after contracting influenza [2]. Neurological complications are reported to occur in 1%–15% of influenza cases in the pediatric age, often self‐resolving, although permanent sequelae or death can occur [3, 4]. In Japan, acute encephalitis and acute encephalopathy in children with influenza have been reported since the 1980s and in various parts of the world [5, 6, 7]. National influenza surveillance data from 2010 to 2015 in Japan showed an average incidence rate as 2.83 per 1 million cases [8]. Other IANCs include acute episodes of chronic neurological conditions, transient visual disturbances, seizures, Reye's syndrome, cerebrovascular accidents, and some rare and delayed complications such as post‐infectious encephalitis, Guillain–Barré syndrome, acute disseminated encephalomyelitis, and transverse myelitis. Currently, there are no statistical reports on neurological damage in children with influenza in Vietnam. The present study aims to describe the clinical and laboratory characteristics of children diagnosed with severe IANCs admitted to the Vietnam National Children's Hospital.

## 2 Materials and Methods

### 2.1 Study Population

A descriptive and prospective study was conducted with 20 patients, aged 1 month–18 years, diagnosed with severe IANCs at the Tropical Pediatric Center–Central Children's Hospital during two influenza seasons from October 2022 to February 2024.

### 2.2 Clinical Case Definitions

Clinical influenza was defined as “a sudden onset of fever, a temperature > 38°C and cough or sore throat in the absence of another diagnosis,” and real‐time reverse transcription polymerase chain reaction (rRT‐PCR) testing detects the influenza virus in respiratory specimens [9].

IANCs are defined as the appearance or exacerbation of neurological symptoms that cannot be explained by other causes in patients diagnosed with influenza based on clinical findings and testing [10].

The diagnosis of encephalitis in children is primarily based on prolonged visual disturbances of at least 24 h, not attributable to other causes, and at least three additional criteria such as fever within 72 h before or after onset, generalized or focal seizures not due to other causes, new focal neurological symptoms, white blood cell count in cerebrospinal fluid over 5 cells/mL, abnormalities on CT/MRI imaging of the central nervous system suggestive of encephalitis, and abnormalities on electroencephalogram suggestive of encephalitis not due to other causes [11].

Influenza‐associated encephalitis occurred when patients met encephalitis criteria and influenza PCR testing in respiratory secretions was positive, while common causes of encephalitis were ruled out.

MRI and CT results in patients with influenza‐associated encephalitis were classified into five groups: normal (category 1); diffuse involvement of the cerebral cortex (category 2); diffuse brain edema (category 3); symmetrical involvement of the thalamus (category 4); and post‐infectious focal encephalitis (category 5) [12].

### 2.3 Data Collection

For patients diagnosed with IANCs, data was collected through the medical history, physical examination, and monitoring, into a predefined medical record template. The collected data included demographic characteristics, clinical features, influenza vaccination status, hospital admission status, complete blood count, acute phase reactants (C‐reactive protein, LDH, and ferritin), serum and cerebrospinal fluid biochemistry, imaging diagnosis results from MRI, CT scans, etiological investigations in serum and cerebrospinal fluid, treatment modalities during hospitalization, and treatment outcomes at discharge.

### 2.4 Statistical Analysis

Statistical analysis was conducted using SPSS 20.0 software. Calculations included mean, median, and percentage values. The correlation was computed using Fisher's exact test.

### 2.5 Ethical Considerations

The included patients were all in the hospital for treatment, and no additional routines or tests were conducted specifically for the study. All participants or legal guardians have given consent for study collection of data, and no individual patient information was collected that could harm the patient.

## 3 Results

The study results from the 20 patients revealed that 16 patients were diagnosed with encephalitis, 3 patients had cerebral infarction, and 1 patient had peripheral facial nerve palsy.

### 3.1 Patient Characteristics

Among the 20 patients, there were 15 (75%) females and 5 (25%) males, with a median age of 39 months (range 12–110), and 16 (80%) were children younger than 5 years old. None of the children received influenza vaccination within the last 12 months. Initial symptoms included high fever (100%), seizures (80%), and altered consciousness (80%). The median time from onset to neurological symptoms was 1 day, with an interquartile range (IQR) of 1–2.75 days. The epidemiological, clinical, and laboratory characteristics of the patients are described in Table 1.

**TABLE 1:** Demographic and clinical characteristics of patients.

Characteristics		*N*	%
Sex	Male	5	25
	Female	15	75
Age (month)	≤ 60 months	16	80
	> 60 months	2	20
Anamnesis	Healthy	14	70
	Chronic health condition	6	30
	Influenza unvaccinated	20	100
Neurologic manifestations	Seizures	16	80
	Altered consciousness	16	80
Fever > 39°C		20	100

Fourteen (70%) patients enrolled in the study had a healthy history, 3 (15%) patients had neurological disorders, 1 (5%) patient was diagnosed with 3‐hydroxy‐3‐methylglutaryl‐CoA lyase deficiency, and 2 (10%) patients developed Moyamoya syndrome after experiencing a stroke.

### 3.2 Laboratory

Most patients showed elevated levels of ALT (60%), AST (90%), LDH (94%), and ferritin (69%) in the serum compared with the reference values within the age group (Table 2).

**TABLE 2:** Admission results from the hematology laboratory.

Characteristics		*N*	%
Leucocyte count (/mL)	Normal	9	45
	Elevated	11	55
ALT (U/L)	Normal	8	40
	Elevated	12	60
AST (U/L)	Normal	2	10
	Elevated	18	90
LDH (U/L) (∑*n* = 16)	Normal	1	6.3
	Elevated	15	93.7
Ferritin (ng/mL) (∑*n* = 16)	Normal	5	31.3
	Elevated	11	68.7
CRP (mg/L)	Normal	10	50
	Elevated	10	50
CSF analysis (∑*n* = 18)	Leucocyte count ≥ 5 cells/mL	4	22.2
	Protein ≥ 0.43 g/L	10	55.6
	Glucose ≤ 2.2 (mmol/L)	1	5.6
Influenza virus typing	Type A	16	80
	Type B	4	20

Abbreviations: ALT, alanine transaminase; AST, aspartate transaminase; CRP, C‐reactive protein; CSF, cerebrospinal fluid; LDH, lactate dehydrogenase.

Eighteen patients underwent lumbar puncture, but the procedure was not performed on two patients with cerebral infarction. Among them, 4 (22.2%) patients had cellular changes, 1 (5.6%) patient had decreased glucose level, and 8 (44.4%) patients had elevated protein level in the cerebrospinal fluid. In all 18 (100%) cases, influenza virus was not detected in the cerebrospinal fluid (Table 2).

Both influenza A and B viruses could cause the disease, with 16 (75%) cases attributed to influenza A virus and 4 (25%) cases to influenza B virus.

### 3.3 Neuroimaging

**FIGURE 1:**
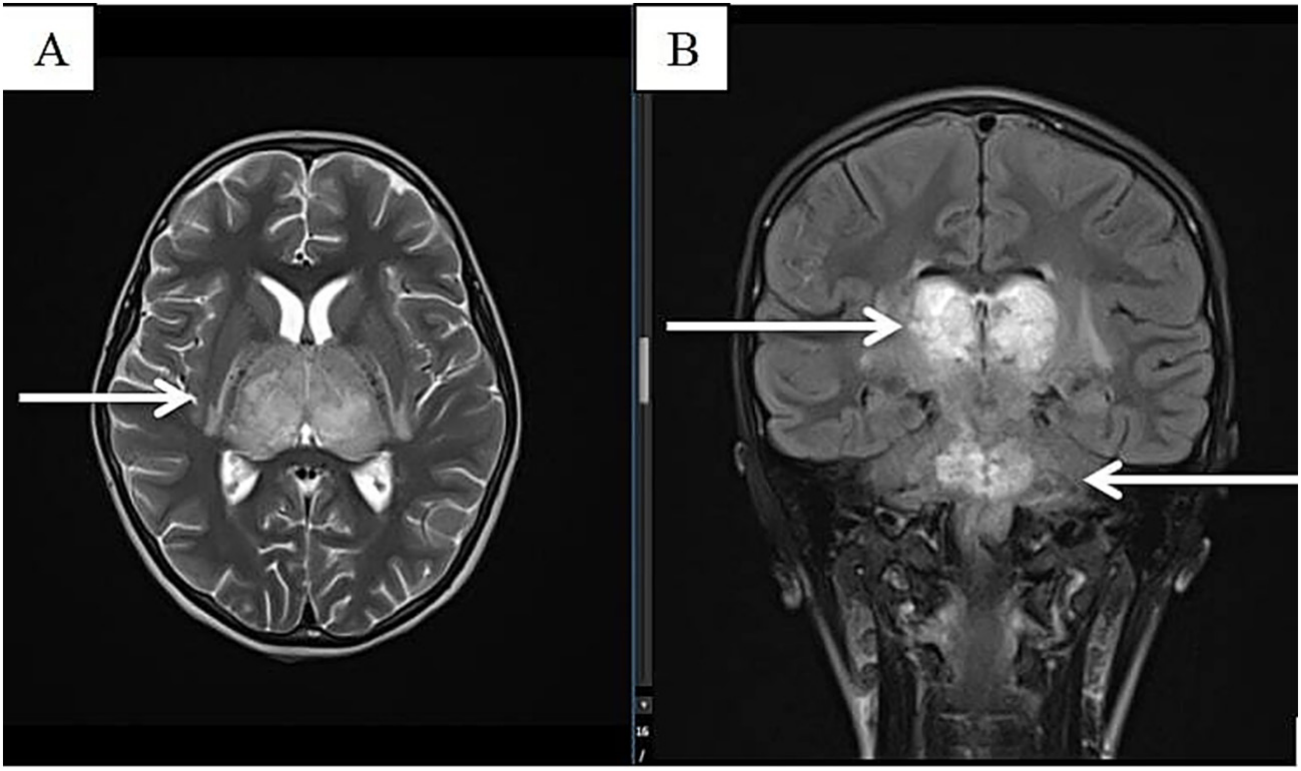
A 7‐year‐old female with bilateral optic nerve damage, involving both the inner and outer sides of both arms, as well as injuries to the cerebellum, both cerebral hemispheres, brainstem, and corpus callosum. (A) T2W sequence; (B) Flair sequence.

**FIGURE 2:**
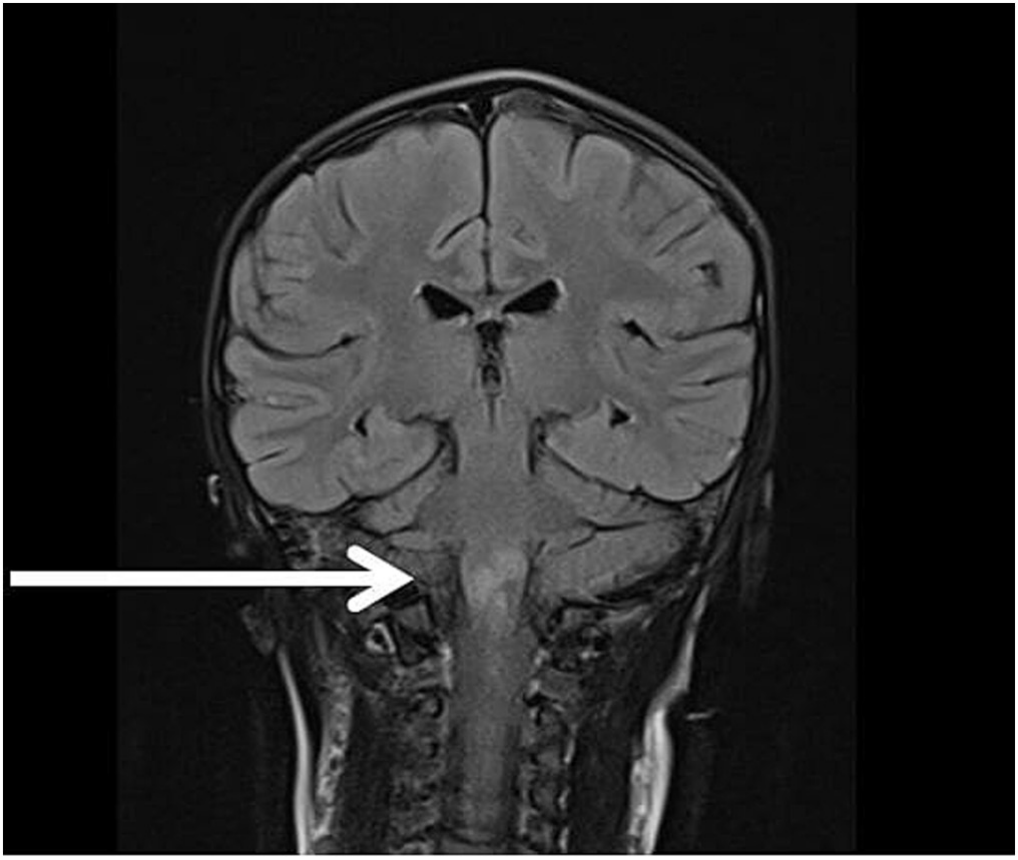
A 5‐year‐old female with brain and cervical spinal cord injuries on Flair imaging.

Eighteen patients underwent computed tomography (CT) or magnetic resonance imaging (MRI) of the brain. Two patients were unable to undergo imaging due to critical conditions and early death after admission. Among them, 3 patients showed signs of cerebral infarction, 1 had normal results, and 13 cases were diagnosed with encephalitis with diverse lesions in the white matter and central gray matter, distributed bilaterally in the cerebral hemispheres (Figure 1), with 1 case showing lesions in the brainstem and upper cervical cord (Figure 2).

### 3.4 Treatments

All 20 patients were treated with oseltamivir, 9 (45%) patients were treated with high‐dose methylprednisolone (30 mg/kg/day × 5 days); 2 (10%) patients received both methylprednisolone and intravenous immunoglobulin (IVIg); 13 (65%) patients required mechanical ventilation. Among the 11 patients treated with methylprednisolone, 3 patients had steroid therapy administered within 48 h after the onset of influenza; however, there was no statistically significant difference in the final treatment outcomes between the two groups (*p* = 0.491). Among the 20 patients in the study, 2 (10%) patients recovered without sequelae, 5 (25%) patients had mild sequelae, 9 (45%) patients had severe sequelae, and 4 (20%) patients died (Table 3).

**TABLE 3:** Treatments and outcome characteristics.

Characteristics		*N*	%
Treatments	IVIg	2	10
	Methylprednisolone	11	55
	Oseltamivir	20	100
	Ventilation	13	65
Outcome	Recovery	16	80
	Death	4	20

Abbreviation: IVIg, intravenous immunoglobulin.

## 4 Discussion

In this study, the influenza associated neurological complications were evaluated in 20 hospitalized children, and the majority of influenza cases with neurological complications occurred in children under 5 years old (80%). Our data were in line with the findings of Donnelley et al. [13] confirming that IANCs are a severe outcome of influenza virus infection, often seen in children with high mortality rates [3]. None of the patients in our study had received seasonal influenza vaccination within the past 12 months. While the study does not provide conclusive evidence that influenza vaccination directly reduces the rate of encephalitis, annual immunization can lower the overall prevalence of influenza infection, thereby indirectly reducing incidence of severe influenza‐related complications, particularly neurological ones. On the other hand, these neurological damages can occur in patients with a healthy medical history; hence, immunizing children against influenza should be encouraged to a higher degree and extent [13, 14].

The majority of IANCs manifested early within approximately 48 h of the onset of influenza symptoms, which is consistent with other reports in the literature [10, 15]. However, there are some rare exceptions, such as Guillain–Barré syndrome, which may occur later. Consistently, this present study revealed that patients experienced neurological symptoms such as seizures and visual disturbances as early as 1 day from the beginning of the illness.

Elevated serum levels of ALT, AST, LDH, and ferritin have been reported to be prognostic factors for the severe condition of influenza patients [16, 17]. Our data showed that majority of patients had elevated serum levels of ALT, AST, LDH, and ferritin (Table 2). A study by Shafran et al. found that elevated liver enzymes are correlated with severity and the risk of death (OR 4.344; 95% CI [2.218, 8.508]) in flu patients [16]. Serum ferritin levels are significantly higher on average in patients with severe pathological conditions. In a multivariate analysis, elevated ferritin levels increase the risk of severe outcomes in influenza patients by five times [17]. Besides its role in intracellular balance, high ferritin levels not only serve as a biological marker of inflammatory response but may also play a role in disease pathogenesis through signaling as part of the innate immune response and regulation of lymphocyte function [18]. Therefore, routine testing of these parameters in severe flu patients can provide clinicians with accurate assessment and prognosis tools for patients with influenza.

The mechanism of nerve damage in influenza is still unclear. Fujimoto et al. reported finding influenza virus RNA in the central nervous system (five out of seven positive cases) [19]. However, some current studies have not detected virus RNA in the cerebrospinal fluid [10, 20]. The increased detection of inflammatory and pre‐inflammatory factors in the blood and cerebrospinal fluid in cases of influenza‐associated encephalitis supports the hypothesis that a “cytokine storm” is a central factor in the pathogenesis of nerve damage in influenza [18, 21, 22, 23]. This hypothesis is consistent with our study findings where most patients had elevated ferritin and LDH levels compared with normal reference values in children; only four patients showed an increase in white blood cells in cerebrospinal fluid, and no cases of flu virus RNA were detected in cerebrospinal fluid.

**FIGURE 3:**
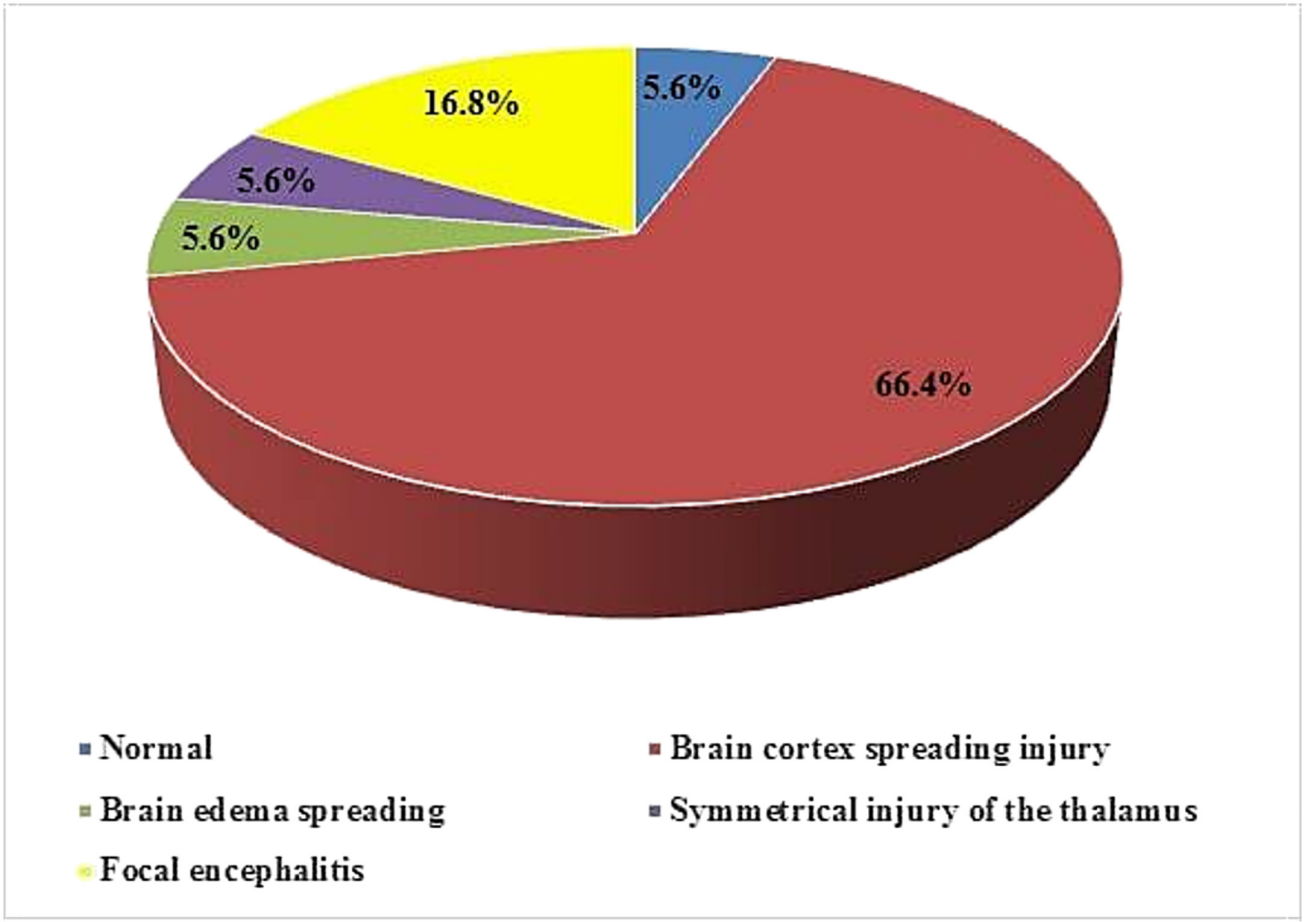
Characteristic of neuroradiological imaging.

Brain imaging findings in patients with brain injury associated with the flu commonly show abnormalities in gray and white matter bilaterally. Kimura et al. categorized MRI brain changes in flu‐related brain injury patients into 5 groups based on MRI and CT results: normal (type 1), brain cortex spreading injury (type 2), brain edema spreading (type 3), symmetrical injury of the thalamus (type 4), and focal encephalitis (type 5) [12]. Figure 3 shows that the majority of patients in our study had type 2 injuries (12 patients), consistent with findings reported in other literature [14, 15, 24].

At present, no specific treatment is available for nerve damage caused by influenza. However, immune therapies, such as corticosteroids or IVIg have been used because of the crucial role of pro‐inflammatory cytokines in promoting the disease process [25]. Early administration of corticosteroids within the first 24 h of neurological symptoms onset may alter the disease progression and improve treatment outcomes [26, 27]. Our study indicated that there was no significant difference in the outcomes between patients had methylprednisolone administered within 48 h after the onset of influenza and the delay steroid therapy group. One possible explanation for this could be the difficulty in identifying nerve damage caused by influenza within the initial 24‐h period, especially in developing countries like Vietnam with limited resources for medical assessment. Consequently, the administration of corticosteroids between 24 and 48 h following the onset of neurological symptoms may not result in a significant reversal of brain damage. Moreover, owing to the study's limited sample size, we could not observe this difference.

It is important to note that our research has certain limitations. First, the complication under investigation is rare; therefore, data collection was performed at a single center, which resulted in a relatively small number of patients being included in the study. This, in turn, may affect the statistical power of the methods used. Furthermore, it should be noted that long‐term outcomes for all patients were not collected as part of this study. As a result, future studies will be required to address these limitations and delve into the underlying mechanisms.

## 5 Conclusions

All children presenting acute neurological features during influenza season should be evaluated for IANCs especially in children under 5 years old. The most common symptoms were altered consciousness, seizures, and increased muscle tone due to damage to the central gray matter, white matter around the ventricles, brainstem, and cerebellum. It is needed to evaluate serum levels of ALT, AST, LDH, and ferritin for better assessment in children with influenza. Treatment requires a combination of various methods; however, the mortality rate and complications remained high.

## Author Contributions

**Sy Duc Nguyen:** conceptualization, writing – original draft, writing – review and editing, methodology, formal analysis. **Thi Huyen Trang Ngo:** data curation, investigation, writing – original draft. **Thi Viet Ha Nguyen:** conceptualization, methodology, writing – review and editing. **Thien Hai Do:** conceptualization, methodology, writing – original draft, writing – review and editing, formal analysis.

## Ethics Statement

This study was approved by the Ethical Review Committee of Vietnam National Children's (approval no. 2661/BVNTW‐HĐĐĐ) and was conducted according to the principle outlined in the Declaration of Helsinki, as well as in compliance with the law concerning the prevention of infections and medical care for patients with infections of Vietnam.

## Conflicts of Interest

The authors declare no conflicts of interest.

### Peer Review

The peer review history for this article is available at https://www.webofscience.com/api/gateway/wos/peer‐review/10.1111/irv.70035.

## Data Availability

The data that support the findings of this study are available from the corresponding author upon reasonable request.
